# Double-level lumbar spondylolysis and spondylolisthesis: A retrospective study

**DOI:** 10.1186/s13018-018-0723-3

**Published:** 2018-03-16

**Authors:** Shengtao Zhang, Conglin Ye, Qi Lai, Xiaolong Yu, Xuqiang Liu, Tao Nie, Haibo Zhan, Min Dai, Bin Zhang

**Affiliations:** 1 Department of Orthopedics, The First Affiliated Hospital of Nanchang University, Artificial Joints Engineering and Technology Research Center of Jiangxi Province, No. 17 Yong Wai Zheng Street, Nanchang, Jiangxi 330006 China; 2 Nanchang University, Nanchang, Jiangxi 330006 China

**Keywords:** Double-level, Spondylolysis, Spondylolisthesis

## Abstract

**Background:**

Lumbar spondylolysis and isthmic spondylolisthesis are common conditions. However, double-level lumbar spondylolysis and spondylolisthesis are rare. We report 24 cases of it along with a review of literature and a briefly description of the clinical and radiological features and integrated management of patients with this condition.

**Methods:**

Of 1700 inpatients diagnosed with lumbar spondylolisthesis at our hospital between January 2008 and September 2015, we selected those with a diagnosis of double-level spondylolisthesis who underwent surgery. We analyzed the data regarding age, sex, and heavy physical labour. Japanese Orthopaedic Association (JOA) and Visual Analog Scale (VAS) scores were used to evaluate preoperative and postoperative neurological function and back pain. All patients underwent decompression, reduction, and posterior lumbar interbody fusion (PLIF) with autogenous bone chips from posterior decompression or with a cage. After the operation, we were followed up for more than 2 years to observe the effect of the operation. In the meantime, the height of the intervertebral discs was measured at follow-up, and all data are analyzed in SPSS stastic.

**Results:**

Double-level spondylolisthesis occurred at the L2/L3 and L3/L4 levels in one patient, L3/4 and L4/L5 levels in 11 patients, and L4/L5 and L5/S1 levels in 12 patients. Nine patients also had spondylolysis. Twenty patients underwent posterior lumbar interbody fusion and internal fixation with autologous bone chip, and 4 of them underwent cage and autogenous bone graft fixation. Postoperatively, the major symptoms (neurological dysfunction and low-back pain) improved significantly. Comparison of JOA and VAS scores indicated effective recovery of neurological function (*p* < 0.05). Postoperative follow-up demonstrated satisfactory interbody fusion and pars interarticularis healing.

**Conclusions:**

Double-level lumbar spondylolysis and spondylolisthesis occurred more often in women. Most common site of double lumbar spondylolisthesis was L3–L5. The treatment principle was the same as that for single-level spondylolisthesis, but the reset order is questionable. Both, posterior lumbar interbody fusion (PLIF) with autogenous bone chips from posterior decompression or with cage can relieve discomfort in most patients. In our follow-up, we found that there was a high degree of loss in disk height when autogenous bone was used. Therefore, we suggest the use of a cage.

## Background

Spondylolisthesis, including degenerative spondylolisthesis (DS) and isthmic spondylolisthesis (IS), is a common degenerative spinal disease and is described as a condition in which a vertebral body, compared to the vertebral body beneath it, shifts forward with an intact neural arch [1–3]. Lumbar spondylolisthesis is seen in 4–6% of the general population [4, 5]. It commonly occurs at the fourth and fifth lumbar vertebrae (L4 and L5) and accounts for more than 95% of the total cases of spondylolisthesis. For a single-segment spondylolisthesis without degenerative disease in the adjacent level, posterior lumbar interbody fusion (PLIF) with pedicle screw fixation is an effective and safe surgical procedure as reported by several papers [6, 7]. However, double-level lumbar spondylolisthesis or lumbar spondylolysis is rare [8–12], and the postoperative results are not similar to those of single segments. We present the data of 24 cases we encountered along with a review of literature. We also briefly describe the clinical and radiological features and the integrated management of patients with this condition.

## Methods

During January 2008 to September 2015, more than 1700 inpatients were diagnosed with lumbar spondylolisthesis at our hospital. Though conservative treatment is commonly the first-line treatment, we adopted surgical intervention in patients whose neurological symptoms such as leg pain and numbness or lumbar back pain were not relieved or exacerbated resulting in an effect on their quality of life. Of these, we identified 24 patients with double-level spondylolisthesis who underwent surgery. Nineteen of them were engaged in heavy physical labour. The mean (± standard deviation, SD) age was 61 ± 8.76 (44–77) years with a mean (± SD) symptom duration of 6.11 ± 6.25 years and a mean (± SD) follow-up of 4.17 ± 1.25 (2–6.58) years. Double-level spondylolisthesis occurred at the L2–L3 level in one patient, L3–L4 level in 11 patients, and L4–L5 level in 12 patients. Nine patients had concomitant spondylolysis. Before the surgery, the patients underwent lateral, flexion, and extension lumbar radiographs; sagittal computed tomography (CT); and magnetic resonance imaging (MRI). Postoperatively, they underwent plain radiography at 1, 3, and 6 months and over 1 year to observe the postoperative effect and fusion rate of the bone graft.

### Preoperative and postoperative assessments

We performed clinical and radiological assessments before and after the surgery. Scores on the visual analog scale (VAS, 0–10 numerical rating scale) and Japanese Orthopaedic Association (JOA) were used to evaluate preoperative and postoperative symptoms and nerve involvement. Additionally, we measured the lumbar intervertebral disc height preoperatively and postoperatively at 1 week, a year, or even longer, in order to observe the changes in the disc height.

## Results

The mean follow-up duration was 4.17 ± 1.25 years. The results are valid because each patient was followed up for at least 2 years. The mean preoperative VAS was 8.88 ± 1.36, while it was 2.25 ± 1.26 postoperatively indicating that pain was relieved effectively (*p* < 0.05). Based on the JOA, the quality of life of the patients has been greatly improved. Bone healing was achieved in 24 patients and signs of root tension were negative in 23. No neurological deficits were noted; pain was not relieved in only one patient. Cage interbody fusion was used in three cases and autogenous bone in 21 patients. The changes in the mean difference of the intervertebral disc height at L3–L4, L4–L5, and L5–S1 were statistically significant (*P* < 0.05) (Table 1). The mean postoperative disc height at 1 year at L3–L4, L4–L5, and L5–S1 was lower than the preoperative values, and significant differences were noted at L4–L5 and L5–S1 (*P* < 0.05) (Table 2). It is proved that the use of autologous bone graft is difficult to maintain the height of the intervertebral disc, hence it is't a good method of bone graft.

**Table 1:** Preoperative (Pre) and postoperative (Po1) lumbar disc height

	*N*	Pre	Po1	*P*
DH (L3/L4)	12	9.34 ± 2.74	11.86 ± 2.48	0.004
DH (L4/L5)	19	8.17 ± 2.12	12.67 ± 2.41	< 0.001
DH (L5/S1)	11	9.64 ± 2.17	11.41 ± 2.46	0.067

**Table 2:** Intervertebral disc height upon follow-up

	*N*	Po 1	Po 2	*P*
DH (L3/L4)	12	11.86 ± 2.48	10.71 ± 2.97	0.100
DH (L4/L5	19	12.67 ± 2.41	10.32 ± 1.74	< 0.001
DH (L5/S1)	11	11.41 ± 2.46	10.76 ± 1.74	0.035

Po 1: The intervertebral disc height was measured within a week of the operation

Po2: The intervertebral disc height was measured within a year of the operation

*N* Perfect number of follow-up patients

### Case report

**Fig. 1:**
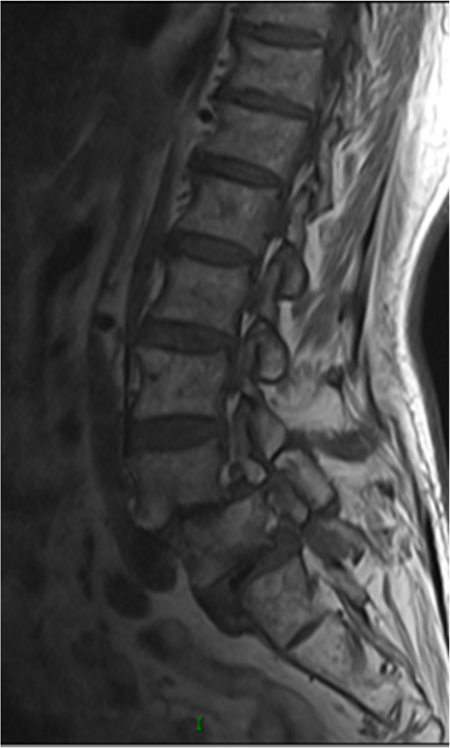
Magnetic resonance image shows II-degree spondylolisthesis at L4 and L5, and the corresponding segment of the nerve compression

**Fig. 2:**
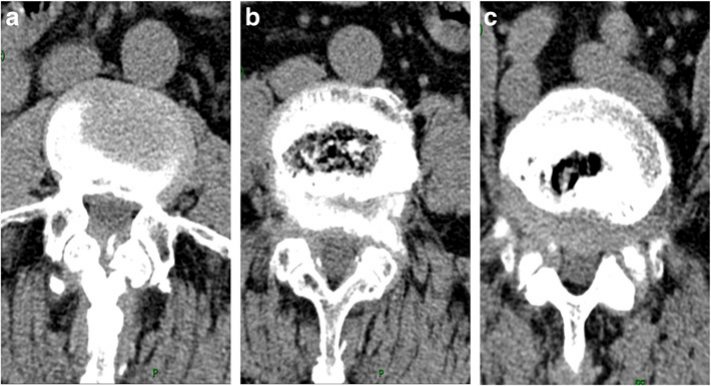
**a**, **b**, **c** Plain computed tomography image of the lumbar intervertebral discs shows L3/4, L4/5, L5/S1 disc herniations, spinal volume reduction, and nerve compression

**Fig. 3:**
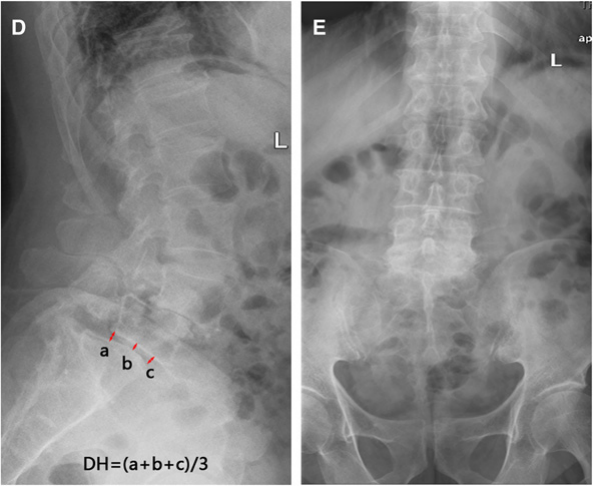
**D**, **E** MRI, CT, X-ray show double-level spondylolisthesis at L4 and L5, in MRI and CT show the corresponding segment of the nerve compression. Preoperative lateral lumbar spine. Slipping of L4 and L5 resulting in II spondylolisthesis, and bilateral spondylolysis at L4 and L5

A 66-year-old man visited our outpatient clinic for severe back pain that he had developed spontaneously 3 years ago, in 2014. However, since it was not associated with significant disability, he was treated conservatively (anti-inflammatory painkillers, back exercises, etc.). Six months before hospitalization, he felt unbearable pain with numbness of the right lower limb and could only walk up to 100 m. On post-admission assessment, VAS was 10 points and JOA 12 points. X-ray, MRI, and CT showed double-level lumbar spondylolisthesis at L4–L5, with bilateral spondylolysis, and significant compression of the corresponding nerve (Figs. 1 and 2). The muscle strength in the right lower limb was grade IV with normal muscle tension and negative straight leg raising test bilaterally. Therefore, we decided to treat him with surgery. We performed PLIF with autogenous bone graft, appropriate reduction, and sufficient decompression of the L4–L5 and L5–S1 vertebrae (Fig. 3). Postoperatively, the back pain and numbness of the lower limbs were significantly reduced without restriction of walking. Before discharge, VAS score was 2 and JOA was 23. At a follow-up after 1 year of self-care and only occasional soreness of the waist and back, VAS score was 1 and JOA score was 25, and imaging examination showed that the lumbar vertebrae reached bony union.

## Discussion

Spondylolisthesis is defined as the anterior or posterior migration, or slippage, of one vertebra in relation to the next caudal vertebra. It mostly occurs in the lumbar spine and is considered to be due to with spondylolysis or degeneration [13]. Spondylolytic spondylolisthesis is distinguished by fracture of the pars interarticularis and is observed primarily during childhood or early adult life [14, 15]. Although the factors for the development of lumbar spondylolisthesis are still not clear, hereditary, traumatic, mechanical, and hormonal factors may play an important role in the process [16]. The occurrence of isthmus may be related to chronic strain, large spine pressure, and large shear force. It is known that women are approximately three times more likely to be affected by spondylolisthesis than men [14, 17, 18], which is consistent with our measurements (6 male/24 patients). Double-level lumbar spondylolysis is more serious spondylolisthesis, as some reports reported that female lumbar spondylolisthesis more easily progress [4]. In this study, we also found that the greater degree of slipping of the L3–L4 and L4–L5 vertebrae promotes easy fracture of the isthmus. It is easy to understand that the greater the angle of slipping, the more unstable the vertebral body is, the easier it is to crack the isthmus. According to VAS and JOA, surgery in most patients with symptoms is effective. Postoperative follow-up CT and X-ray showed good bone fusion at 1 year; therefore, both the surgical methods can be effective at improving the symptoms.

**Fig. 4:**
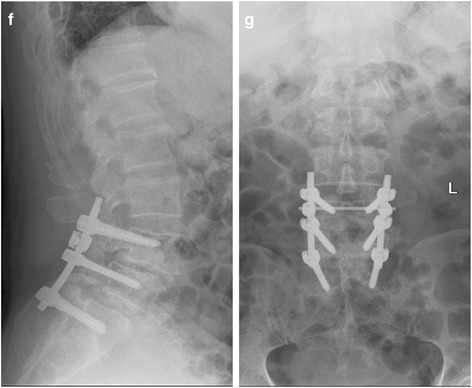
**f**, **g** Postoperative lumbar spine showing L4–L5 correction with restoration of the physiological curvature of the spine

Although spondylolisthesis is usually asymptomatic, it can progress to spinal stenosis and result in neurogenic symptoms, such as leg pain, numbness, or weakness [19]. Conservative treatment is the first consideration, but if regular conservative treatment has not been alleviated and the quality of life is affected, surgery is an effective solution. Treatment included a decompression alone, decompression and fusion, fusion including postero-lateral, interlaminar fusion, and interbody fusion. For two-level spondylolisthesis patients, spinal stability is poor and needs to be fixed and fused; at the same time, we adopted the most biomechanical interbody fixation. So surgery in such cases is mainly for lumbar fixation, restoration of physiological curvature, and decompression of the compressed nerve root (Fig. 4). Despite a variety of surgical methods as previously reported [20], there are five main approaches: posterior lumbar interbody fusion (PLIF), transforaminal lumbar interbody fusion (TLIF or MI-TLIF), oblique lumbar interbody fusion/anterior to psoas (OLIF/ATP), anterior lumbar interbody fusion (ALIF), and lateral lumbar interbody fusion (LLIF). The advantages of ALIF are that it retains all the posterior-stabilizing structures, reduces adjacent segment disease from denervation or injury to the adjacent facet joints and muscles, and avoids, both, epidural scarring and perineural fibrosis [21]. However, its risks include a potential for visceral injury (5%), retrograde ejaculation and sympathetic dysfunction (3%) [22], and difficulty with revision due to the potential for scar tissue formation on the interface between the aorta and common iliac vein on the anterior border of the spine [23]. In our study, we adopt PLIF because it allows for adequate interbody height restoration and neural decompression while maintaining posterior support structures, [24]. After posterior pedicle screw fixation, most of the upper vertebral body force was transferred to the lower vertebral body through the nail bar system. The bone conduction and internal pressure decreased significantly, which greatly reduced the incidence of pedicle screw loosening and fracture. Surgical decompression mainly includes the spinous process and the whole laminectomy, the medial part of the upper and inferior articular processes, nerve root canal scar tissue resection, and strive to complete decompression. The intraoperative technique is mainly to accomplish moderate reduction, not necessarily completely reset, because excessive reduction will also cause nerve root compression because of the soft tissue card. On the contrary, resetting can eliminate the spondylolisthesis and restore the normal physiological curvature of the lumbar spine, thereby improving the appearance of the lumbar spine and ensuring the normal physiological line of the lumbar spine. Spondylolisthesis can lead to narrowing of the intervertebral foramen, and resetting of the vertebral body can restore the height of the intervertebral foramen, thus reducing the nerve root compression symptoms caused by the stenosis. In 23 patients, pain in the waist and back, and numbness and pain in the lower extremities disappeared or improved markedly after the surgery, and 1 case was not relieved. The success rate of interbody fusion was 87.5% (21/24), and there are no screw breakage and slippage phenomenon during the follow-up period.

**Fig. 5:**
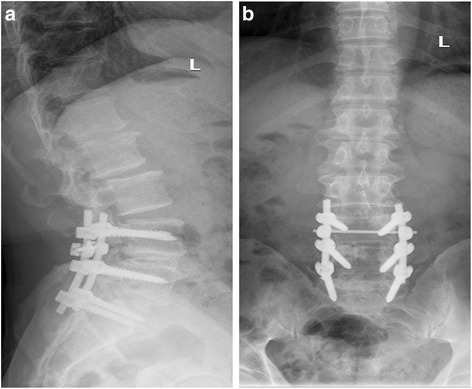
**a**, **b** No spondylolisthesis was seen after 1 year, and bone healing was achieved in the intervertebral bone graft

In earlier days, we used autologous bone fragments, and later on, cage was used. It has been reported that changes in disc height between the pre- and postoperative periods were significant with autologous bone and cage [25]. Based on the disc height measurements, we can see that whether interbody fusion with autogenous bone chips from posterior decompression or with cage, after pulling nail rod reduction, intraoperative distraction, disc height can be restored, but resulting in postoperative loss of disc height, statistics show that there are differences (*P* < 0.05). It may have several reasons: first, cage can provide an immediate stability, and second, during the process of bone fusion, bone chips undergo dissolution, absorption, cell growth, and trabecular bone absorption. Under the biological pressure, the height of intervertebral disc will decrease, and using cage fusion device may help maintain the height of intervertebral disc. In conclusion, the use of cage intervertebral bone graft is more helpful to maintain the height of the intervertebral disc (Fig. 5).

The reduction order is worth discussing. According to the direction of slip, multi-segment degeneration slippage is divided into three categories: the former spondylolisthesis, posterior spondylolisthesis, and mixed spondylolisthesis. Clinically, the most common are the former and mixed spondylolistheses. Due to different directions of vertebral spondylolistheses, reduction of multi-segment involvement is more difficult than that of a single segment. Intraoperatively, we found that due to the instability of adjacent vertebral bodies, the lower spondylolisthesis cannot provide mechanical fulcrum and the upper vertebral spondylolisthesis aggravated. In order to make the reduction simple, the “fulcrum” vertebral body was reset first in the operation according to the type of slipping. We suggest that according to our experience, anterior spondylolisthesis should be first reset, followed by fixation of the superior vertebral body and reattachment of the lower vertebral body. After the upper vertebral reduction, the lower vertebral body is repositioned through the upper metal rod. Mixed slippage while fixing the upper and lower vertebral bodies, and then synchronize lifting intermediate vertebrae slippage, may be reset from the bottom to the slide and fixed to the lower vertebral body, and then reset, the upper slide fixing vertebrae. While fixing difficulties, pedicle pulling system, pre-bent rods titanium leverage to reset the instrument.

There are certain limitations to our study. We adopt dynamic lateral flexion and extension X-ray films to evaluate the fusion state, which is widely used. However, CT is more advantageous in evaluating the imaging diagnosis of spinal fusion. Because of the low incidence rate, the number of single centre samples is small and the conclusions are limited.

## Conclusion

In conclusion, we have reported 24 cases of double-level lumbar spondylolisthesis and have summarized the results of a literature review. We found that both surgical techniques could significantly improve pain and disability in patients with double-level lumbar spondylolisthesis and achieve good mid-term prognosis. We compared autologous bones and cages and provided some thoughts on vertebral resuscitation. Further quantitative and detailed studies are required in this topic.

## Abbreviations

ALIF: Anterior lumbar interbody fusion
CT: Computed tomography
JOA: Japanese Orthopaedic Association
PLIF: Posterior lumbar interbody fusion
Po: Postoperative
Pro: Preoperative
VAS: Visual Analog Scale
